# On Nephropexy

**Published:** 1913-09

**Authors:** J. Lacy Firth

**Affiliations:** Surgeon to the Bristol General Hospital.


### ON NEPHROPEXY.

BY

J. Lacy Firth, M.D., M.S. (Lond.), F.R.C.S.,
*Surgeon to the Bristol General Hospital.*

---

My object in this communication is a double one—to express appreciation of the operative technique in nephropexy recommended by William Billington, and at the same time to emphasize the fact that in the operation great caution has to be taken to avoid wounding the pleura. I also record a case in which this accident occurred in my practice.

It was in November, 1910, that I decided to try the method of Billington[1] for fixing a movable kidney. For several years previously, following Jonathan Hutchinson, jun., I had been fixing kidneys with kangaroo tendon sutures passed through the organ and through the muscular edges of the wound on each side. Earlier still I had practised the method of Edebohls, in which the renal capsule is deflected from both the anterior and posterior surfaces of the kidney half-way to the hilum, and the deflected portion of the capsule and the undeflected part nearby are transfixed with fixation or suspension sutures, the ends of which are carried through the muscles on each side of the wound. My reason for changing from Edebohl's method to Hutchinson's was the feeling that it was a mistake to decapsulate the kidney, because decapsulation seemed unnecessary, and perhaps caused some atrophy of the renal cortex. It appears to me to-day that partial or even complete decapsulation of the kidney is a procedure which leads to no appreciable evil results, and that it is the one procedure which most certainly secures firm adhesion between the kidney and surrounding structures. In this connection I may allude to the experiments on cats

[1] *Movable Kidney*, by William Billington, M.S., F.R.C.S. Cassell and Co. 1910.

made by Harold W. Wilson and C. M. Hinds Howell.[1] These writers, comparing the results of suturing without decapsulation and suturing after partial decapsulation, say, "We can confidently assert that that of decapsulation secures by far the more efficient fixation."

My reason for passing to Billington's method after using the others described was that by it the kidney is fixed at a normal level, whereas in the other two the kidney is fixed at a level considerably lower than normal.

Writers have alleged that no harm results from fixing the kidney in a lower position than normal, if the fixation holds good. Others have reported troubles which seemed to be due to the low position in which the organ had been fixed. One of my patients in whom I had fixed the kidney rather low, after an interval of six months of relief, began to complain of pain in the corresponding lumbar region of the abdomen, and she continued to complain for eighteen months or more. At last, in desperation, I determined to try and refix the kidney at a higher level. I found, however, that the kidney was so firmly adherent that it was impossible to improve its position, and I had to give up the attempt. I cannot be at all certain that this patient's pain was due to the low position of the kidney. She is a person never without pain somewhere. Before her kidney had been touched she had had a more or less normal appendix removed for pain simulating appendicitis.

The above is the only example I remember seeing of trouble due, or probably due, to fixation of the kidney in too low a position. Billington,[2] after quoting with approval Gœlet's statements that "fixation of the kidney lower than normal is worse than mobility," and that "this is what produces the great percentage of failures," states that he himself has several times had to operate upon kidneys previously fixed too low, and which were causing severe pain.

Anatomical considerations suggest that a fixation of the

[1] *Movable Kidney*, by Harold W. Wilson and C. M. Hinds Howell. London: Edward Arnold, 1908, p. 89.

[2] *Op. cit.*, p. 149.

kidney in a low position may result in kinking the ureter, and cases of hydronephrosis following renal fixation have been attributed to this state of affairs.

On the other hand, some writers have recommended fixing the kidney lower than usual to avoid the exertion of pressure upon it by the liver, specially during inspiration, which pressure tends to displace the organ again, or to flex its upper pole. Fixation of the kidney to the last rib has been particularly objected to, because the normal movement of the last rib is upwards with inspiration. The kidney should move slightly downwards with inspiration to avoid diaphragmatic pressure.

My first impressions on reading Billington's description of the operation he uses were that the method was condemned by its meddlesomeness and complexity. But I have found it easy to perform, and not so complicated as its description might lead one to suppose.

For anything like a complete description of the operation here labelled Billington's I must refer the reader to that surgeon's original writings. He lays no "claim to originality in connection with any particular step in the operation." Methods and principles suggested by Edebohls, Fullerton, Gœlet and Brödel have been combined in the operation, and the combination of methods adopted seems to have been first used by Jordan Lloyd.

Briefly outlined, the following are the steps of the operation: (1) Oblique incision, beginning over the eleventh intercostal space; (2) separation of the fat and perinephric fascia "from the muscles of the back for some distance above and below the last rib"; (3) dislocation of the kidney into the loin, and separation of all adhesions and fat; (4) deflection of a flap of renal-capsule downwards from the upper half of the kidney, two-thirds of the flap being from the posterior surface, one-third from the anterior; (5) insertion of two supporting sub-capsular sutures, after the manner of Gœlet and Brödel, into the lower half of the kidney, the ends of these being long to pass through the muscles and skin above the wound to be tied over gauze rolls; (6) passing a curved Spencer Wells forceps through the eleventh intercostal space at the edge of the erector spinæ muscle, so that the ends of the forceps curve round the last rib and project below into the upper part of the wound; and (7) passing the capsular flap, grasped in the forceps mentioned, over the last rib and suturing the portion drawn out down to the capsular surface of the kidney at the lower border of the rib.

Billington's assertion that, by the method of operating outlined above, the kidney can be fixed in a normal position is based upon investigations made by him in conjunction with Professor Arthur Robinson. He writes[1]: "Dissections of formalin-hardened bodies were made. It was found, with great constancy, that in the normally-placed kidney the angle formed by the last rib and the outer edge of the erector spinæ corresponds to a point on the posterior surface of the kidney midway between the upper and lower poles, and about half-way between the hilum and convex border." This middle point of the posterior surface of the movable kidney can be brought almost exactly to the other corresponding point, that is to the angle of the last rib and erector spinæ, by the operation described. The capsular flap in the operation, as pointed out above, is detached downwards only to the level of a line midway between the upper and lower poles of the kidney. In other words, the flap remains attached to the kidney by its base-line at this level, and the base-line is brought into contact with the last rib, covered by the diaphragm, when the flap is drawn taut over the rib. The kidney need not be placed so high. If the liver has a lower position than normal, and encroaches upon the space which is ordinarily filled by the upper pole of the kidney, it will be wiser to fix the kidney in a position a little lower to avoid downward pressure.

The most serious drawback of the Billington operation, to my mind, is the danger it entails of wounding the pleura when a channel is being made over and beneath the last rib. I have performed the operation ten times. On each occasion, when taking this step in the operation, I have been afraid that

[1] *Op. cit.*, p. 149.

I might hear that unpleasant hissing sound which is made when air enters the pleural cavity with inspiration through a wounded pleura. At the sixth operation I had the mortification of hearing that sound. I give further details and the subsequent history of the case below. Such an occurrence immediately suggests the question whether this step in the operation is worth taking or not. The uppermost suspensory Brödel suture brings the base-line of the flap and the kidney to the proper level, and retains them there for three weeks. Is not that sufficient ? If not, the fixing power of the flap then comes into play, for the suspensory silk-gut sutures are removed. For the first three weeks I consider the capsular flap to be almost useless.

On the subject of the relations of the pleura to the seat of operation and the danger of wounding it Billington makes the following remarks :[1] "Usually the pleura does not extend below the lower border of the last rib, and for one and a half inches from the tip it terminates at or above the upper border. In some cases, however, the pleura extends lower." And again : "The only precautions necessary are to use a blunt instrument, to keep close to the rib, and to make the puncture not more than one and a half inches behind the tip. I have executed this manœuvre upwards of 350 times, and have only opened the pleura once."

Before my sixth operation the above statements gave me considerable confidence in my ability to avoid wounding the pleura, but since the accident hope has had to replace confidence. My hope for the future is the greater because the instrument I used for clearing a space around the rib was not sufficiently blunt. I was not in possession of a curved Spencer Wells forceps, the instrument recommended by Billington, so I used an aneurysm needle. It is quite likely I should have safe-guarded the pleura if I had used blunt forceps.

There are, however, facts which show that the pleura is liable to puncture in any operation for exposure of the kidney by the lumbar route if the deeper part of the incision at its

[1] *Op. cit.*, p. 3.

upper end is carried to or nearly to the lower border of the last rib, or to the rib which is supposed to be the last one. Thus in Jacobson and Rowlands's *Operative Surgery*,[1] a case is mentioned in which the accident occurred because the last rib was rudimentary, and the pleura came down well below the eleventh rib. The moral to be drawn from that case is that the ribs should be counted from above. Also Lange is quoted as having shown that in exceptional cases the pleura may descend considerably below a normally developed last rib. I have myself seen the pleura opened in exposing the kidney apparently because the pleura descended below the last rib.

The case I report below appears to me worth recording, not only as a warning of the danger under discussion, but also because it illustrates the possible effects of the accident. It will be noticed that dyspnœa gradually increased for two days, but was not noticeable for several hours after conclusion of the operation. These facts suggest that air continued to enter the pleura for many hours. How that could happen is difficult to understand. There was a drainage tube, a small one, in the lower part of the wound, but this was some inches below the last rib, and the kidney lay vertically between the tube and pleural wound, and of course there was a large dressing over the wound.

The following are notes of the case :—

Miss L., aged 23, had a very mobile right kidney, and suffered much from dyspeptic symptoms. A gastric ulcer had been suspected to exist. On October 31st, 1912, the kidney was exposed, brought out onto the loin and all adhesions separated. The capsule was deflected from the upper half of the kidney in the way described above. The last rib was then cleared at the border of the erector spinæ, an aneurysm needle being the instrument used for the purpose. As the needle end appeared in the upper part of the wound at the lower border of the last rib air began to enter the pleura. Mops were immediately applied to the spot to prevent further entry of air, and it was decided to cut away the capsular flap and trust entirely to the two supporting silkworm-gut sutures to retain the kidney in

[1] *The Operations of Surgery*, by W. H. A. Jacobson and R. P. Rowlands. Fifth Edition. Vol. ii. 172.

place. The lower half of the kidney was smeared with pure carbolic acid. As soon as the kidney was drawn up into position by the sutures all suction of air into the pleura seemed to cease. No effects from the air entry could be detected either by the anæsthetist or by me, and my impression was that very little air had entered the pleura. A small drainage tube was placed below the kidney, and the wound closed in layers. I saw the patient again six or seven hours after operation. She was in some pain, but appeared to have no respiratory trouble. At the end of twenty-four hours, on November 1st, she complained of some difficulty in breathing, and there were the physical signs of a pneumo-thorax over the front of the right chest, and the heart's apex beat was about an inch outside the nipple line on the left.

November 2nd (forty-eight hours after operation).—Difficulty in breathing more marked, respirations rapid, patient rather dusky in appearance, resonance on percussion from the right chest across the middle line to slightly beyond the left edge of the sternum in the cardiac region. The apex beat was still further displaced to the left.

An aspirator needle was therefore passed into the chest on the right side through the seventh intercostal space near the posterior axillary line, and connected with the vacuum made in a Winchester quart bottle. Air immediately passed into the bottle from the chest. At the same moment the patient began to have a series of short coughs, rapidly repeated, and she became more dusky and distressed. The vacuum was shut off, and after a pause of half a minute or a minute the cough ceased, and the communication with the bottle was re-established. More air came from the chest and more coughing occurred, but not so much as before. The vacuum was again shut off, and after a pause again connected with the chest, and then the needle withdrawn, as no more air seemed to be obtainable from the pleural cavity. The signs of pneumo-thorax could not now be elicited, and the apex beat had come back at least an inch nearer its normal position. The patient would not confess that her breathing was easier for another ten minutes. At that time there was no doubt possible but that she was considerably relieved of her respiratory distress. From this time onwards the course of convalescence was normal.

In conclusion, let me say that so far as I can judge on theoretical grounds, and as a result of a very limited practical experience, Billington's operation for movable kidney is a very good one, perhaps the best yet described.

